# Pulmonary miRNA expression after polytrauma depends on the surgical invasiveness and displays an anti-inflammatory pattern by the combined inhibition of C5 and CD14

**DOI:** 10.3389/fimmu.2024.1402571

**Published:** 2024-08-29

**Authors:** Nan Zhou, Rald V. M. Groven, Klemens Horst, Ümit Mert, Johannes Greven, Tom Eirik Mollnes, Markus Huber-Lang, Martijn van Griensven, Frank Hildebrand, Elizabeth R. Balmayor

**Affiliations:** ^1^ Experimental Orthopaedics and Trauma Surgery, Department of Orthopaedics, Trauma and Reconstructive Surgery, University Hospital Rheinisch-Westfälische Technische Hochschule (RWTH) Aachen, Aachen, Germany; ^2^ Department of Cell Biology-Inspired Tissue Engineering, MERLN Institute for Technology-Inspired Regenerative Medicine, Maastricht University, Maastricht, Netherlands; ^3^ Division of Trauma Surgery, Department of Surgery, Maastricht University Medical Center+, Maastricht, Netherlands; ^4^ Department of Orthopaedics, Trauma and Reconstructive Surgery, University Hospital Rheinisch-Westfälische Technische Hochschule (RWTH) Aachen, Aachen, Germany; ^5^ Research Laboratory, Nordland Hospital Bodø, Bodø, Norway; ^6^ Department of Immunology, Oslo University Hospital, and University of Oslo, Oslo, Norway; ^7^ Institute of Clinical and Experimental Trauma Immunology, University Hospital Ulm, Ulm, Germany

**Keywords:** respiratory failure, polytrauma, microRNAs, ARDS, early total care, damage control orthopedics

## Abstract

**Background:**

Respiratory failure can be a severe complication after polytrauma. Extensive systemic inflammation due to surgical interventions, as well as exacerbated post-traumatic immune responses influence the occurrence and progression of respiratory failure. This study investigated the effect of different surgical treatment modalities as well as combined inhibition of the complement component C5 and the toll-like receptor molecule CD14 (C5/CD14 inhibition) on the pulmonary microRNA (miRNA) signature after polytrauma, using a translational porcine polytrauma model.

**Methods:**

After induction of general anesthesia, animals were subjected to polytrauma, consisting of blunt chest trauma, bilateral femur fractures, hemorrhagic shock, and liver laceration. One sham group (n=6) and three treatment groups were defined; Early Total Care (ETC, n=8), Damage Control Orthopedics (DCO, n=8), and ETC + C5/CD14 inhibition (n=4). Animals were medically and operatively stabilized, and treated in an ICU setting for 72 h. Lung tissue was sampled, miRNAs were isolated, transcribed, and pooled for qPCR array analyses, followed by validation in the individual animal population. Lastly, mRNA target prediction was performed followed by functional enrichment analyses.

**Results:**

The miRNA arrays identified six significantly deregulated miRNAs in lung tissue. In the DCO group, miR-129, miR-192, miR-194, miR-382, and miR-503 were significantly upregulated compared to the ETC group. The miRNA expression profiles in the ETC + C5/CD14 inhibition group approximated those of the DCO group. Bioinformatic analysis revealed mRNA targets and signaling pathways related to alveolar edema, pulmonary fibrosis, inflammation response, and leukocytes recruitment. Collectively, the DCO group, as well as the ETC + C5/CD14 inhibition group, revealed more anti-inflammatory and regenerative miRNA expression profiles.

**Conclusion:**

This study showed that reduced surgical invasiveness and combining ETC with C5/CD14 inhibition can contribute to the reduction of pulmonary complications.

## 1 Introduction

Polytrauma patients can suffer from severe post-traumatic complications, including sepsis, acute renal failure, Systemic Inflammatory Response Syndrome (SIRS), Compensatory Anti-inflammatory Response Syndrome (CARS), Multiple Organ Dysfunction Syndrome (MODS), and Acute Respiratory Distress Syndrome (ARDS) (1). With documented mortality rates of up to 40%, ARDS has historically been a leading cause of trauma-related morbidity and mortality (2). In part, this is because the management of ARDS can be difficult, with limited effective treatment options other than supportive mechanical ventilation (3). Identifying factors that are important in the occurrence and progression of ARDS is therefore important to improve its treatment as an integral part of polytrauma management.

After the trauma itself, the subsequent surgical trauma is often referred to as “the second hit” (4). Two well-known trauma treatment strategies are Early Total Care (ETC) and Damage Control Orthopedics (DCO), mainly differing in the surgical invasiveness (5). The main principle of ETC is to definitively fixate long-bone fractures during primary fracture surgery, enabling rapid patient mobilization. DCO aims at temporary fracture fixation to stabilize the patient prior to definitive surgical fixation, requiring follow-up surgeries and prolonging hospitalization time. It is known that the type of surgical treatment influences the incidence of pulmonary complications (6). For example, ETC treatment has been associated with reduced pulmonary complications and shorter in-hospital stays in a specific group of patients compared to delayed stabilization (7). However, for severely polytraumatized patients, not every patient is physiologically stable enough to undergo such invasive surgery, and may, therefore, develop previously mentioned complications such as ARDS, SIRS, CARS, and MODS (8). In such a case, DCO allows for the primary restoration of immunological homeostasis prior to more invasive surgery. In clinical practice, the Safe Definitive Surgery (SDS) concept involves the repeated assessment of a patient to dynamically apply the ETC and DCO principles (9).

Together with the applied surgical treatment, the post-traumatic immune response is of importance for the incidence of respiratory complications after polytrauma. For unstable patients with life-threatening injuries, increased surgical trauma, such as in ETC, can exacerbate systemic inflammation by triggering immune responses to a greater extent as compared to DCO (10). Intervening with this post-traumatic immune response may therefore be a good therapeutic approach. A key component in this immune response is the early activation of the complement system. The traumatic release of endogenous factors, such as Damage-Associated Molecular Patterns (DAMPs), leads to the rapid activation of the complement system with release of inflammatory activation products like C3a and C5a. These factors trigger the production of various inflammatory cytokines and contribute to an enhanced inflammatory load (11). When focusing specifically on the lung, research has also shown that the invasion of C5a-activated neutrophils in the pulmonary parenchyma causes necrosis of vascular endothelial cells, which leads to ARDS (12). Furthermore, engagement of CD14, a main co-receptor for several toll-like receptors (TLR) including TLR4 and TLR2, expressed by granulocytes and monocytes/macrophages, triggers the release of proinflammatory mediators and chemokines by immune cells, contributing to the development of pulmonary inflammation (13). Therefore, applying a combined inhibition therapy targeting complement factor C5 and the CD14 (C5/CD14 inhibition) is of interest since their simultaneous inhibition improved the outcome of sepsis by reducing the expression of inflammatory cytokines, such as IL-1β, IL-6, TNF and CXCL1 (14). Although research has been done on the differentiating pulmonary effects of the two surgical treatment strategies ETC and DCO, exact biomolecular mechanisms that may contribute to the incidence of ARDS are not fully known and understood. In particular, the possible ameliorating effects of the C5/CD14 inhibition on pulmonary outcome has not been investigated.

At a biomolecular level, the expression of tissue-specific, regenerative, and immunological proteins is in part influenced by microRNAs (miRNAs). MiRNAs are noncoding RNAs of 21–25 nucleotides in length that can bind or directly cleave a target messenger RNA (mRNA). Thereby, miRNAs prevent mRNA translation into a given protein. Furthermore, miRNAs can enhance the transcription of specific proteins by enhancing their promotor activity (15). It is well known that miRNAs are involved in a wide range of patho-, immuno-, and physiological processes (16). MiRNAs may therefore also play key roles in polytrauma, and more specifically in its crosstalk with ARDS. Some studies have shown correlations between the expression of circulating miRNAs and pulmonary complications, blunt chest trauma severity, as well as overall mortality in polytrauma patients (17, 18). However, research on the exact role and involvement of miRNAs in polytrauma linked to ARDS is scarce.

The two-fold aim of this study was therefore to first determine pulmonary miRNA expression profiles after polytrauma, comparing the two different surgical treatment strategies ETC and DCO. Second, due to certain complications associated with the application of ETC in severely polytraumatized patients, a third treatment group received ETC combined with a novel, combined inhibition therapy, C5/CD14 inhibition, aiming at reducing the inflammatory load after trauma and surgery. For all treatment groups, mRNA targets of differentially expressed miRNAs were bioinformatically predicted to gain insights into the regulated biomolecular pathways concerning polytrauma.

## 2 Materials and methods

### 2.1 Animal care

The German governmental office of Animal Care and Use, LANUV, of the Ministry for Nature, Environment and Consumer Protection in the North Rhine-Westphalia province, reviewed and approved the applied study protocols and procedures in compliance with German legislation under permit number 81.02.04.2020.A215. The study was designed and performed following the 3R principles (Replace, Refine, Reduce). This manuscript is written in adherence to the ARRIVE Guidelines for reporting animal research (19). The study presented here was part of a larger project (20). However, this study was determined *a priori* and the data presented here are original to this publication.

### 2.2 Polytrauma induction

The porcine polytrauma model used in this study has been described in detail elsewhere (20–22). For acclimatization purposes, male German landrace pigs (*Sus scrofa*), aged three months and with a mean body weight (BW) of 35 ± 5 kg, were housed in ventilated rooms seven days prior to polytrauma induction. During the 12-h fasting prior to the experiment, water was available to the animals *ad libitum*. Azaperone (3-4 mg/kgBW, Stresnil™, Janssen-Cilag GmbH, Neuss, Germany) and ketamine (15mg/kgBW, Ketanest^®^, Pfizer, New York, NY, USA) was administered intramuscularly as premedication. After induction of general anesthesia with propofol (1-2 mg/kgBW, Fresenius SE & Co. KGaA, Bad Homburg, Germany), orotracheal intubation was performed and animals were mechanically ventilated with a Draeger Evita 4 (Draeger Safety AG & Co. KGaA, Lübeck, Germany). Volume-controlled ventilation parameters were as follows, tidal volume of 8-12 ml/kgBW, peak end expiratory pressure of 8 mmHg, and a plateau pressure < 28 mmHg. Throughout the whole experiment, vital signs were recorded, and animals were kept under general anesthesia using propofol (5-10 mg/kgBW/h, Fresenius SE & Co. KGaA), fentanyl (1 μg/kgBW/h, Panpharma GmbH, Trittau, Germany), and midazolam (0.03-0.2 mg/kgBW/h, Panpharma GmbH).

A total of 26 animals were randomly allocated into four groups, namely sham, DCO, ETC, and ETC + C5/CD14 inhibition. The sham group, which didn’t receive a therapeutic intervention, was maintained at n=6 leveraging our historical data on this model (20–22) and complying with the 3R guidelines of laboratory animal research (Replace, Reduce, and Refine). One out of 8 animals in the ETC group did not survive until the 72 h time of observation for causes unrelated to the study. The ETC + C5/CD14 inhibition group included n=4 animals. This group was conducted as a pilot study given the limited availability of the inhibition drug. Nevertheless, this pilot group allowed us to investigate the potential value of this drug in the context of polytraumatized, critically ill large animals. Thereby, the final animal allocation was as follows, 1) sham (n=6), 2) DCO (n=8), 3) ETC (n=8), 4) ETC + C5/CD14 inhibition (n=4).

The sham group received identical anesthesia, mechanical ventilation, nutrition, and vasopressor treatment if required. The other three treatment groups were subjected to a standardized polytrauma (Injury Severity Score, ISS=27), consisting of blunt chest trauma, bilateral femur shaft fractures, a crosswise liver incision, and hemorrhagic shock. Throughout polytrauma induction and the 90 min shock phase, FiO_2_ was set to 0.21 to mimic ambient air. A bolt gun machine (turbocut Jopp GmbH, Bad Neustadt, Germany) was used to hit a pair of custom-made panels (steel 8 mm and lead 10 mm) located on the right dorsal, lower chest, resulting in blunt chest trauma. By hitting a special punch with the bolt gun machine on the middle thirds of the femora, bilateral femur fractures were induced. Additionally, the left liver lobe was exposed through a mid-line laparotomy, and two crosswise incisions (4.5 x 4.5 cm) were made halfway through the tissue to mimic liver laceration. After allowing the wound to bleed uncontrolled for 30 seconds, it was covered with 10 × 10 cm sterile gauze. Immediately thereafter, a pressure-controlled hemorrhagic shock was performed by withdrawing blood until the mean arterial pressure (MAP) was equal to 40 ± 5 mmHg, maximally withdrawing 45% of the total blood volume, and left untreated for 90 min to mimic real-life circumstances. Subsequently, the animals were resuscitated following ATLS and AWMF-S3 guidelines (ATLS^®^, AWMF-S3 guideline on Treatment of Patients with Severe and Multiple Injuries^®^). In brief, fluids were administered (Sterofundin^®^ ISO, 2 ml/kgBW/h, B Braun SE, Melsungen, Germany), the withdrawn blood was reinfused (citrate-phosphate-dextrose-adenine anticoagulant mixture, DONOpacks, Lmb Technologie GmbH, Schwaig, Germany), and the fraction of inspired oxygen, FiO_2_, was increased to 0.3 (23). Normothermia was guaranteed using hot air blowers and blankets.

After polytrauma induction and treatment, the animals were monitored in an intensive care unit (ICU) setting for 72 h before sacrifice. Prior to surgery and for every following 24 h, intravenous infusions of ceftriaxone (2 g, Fresenius SE & Co. KGaA) were administered. Meanwhile, animals received parenteral nutrition (Aminoven, Fresenius Kabi Deutschland GmbH, Bad Homburg, Germany) for 72 h while receiving fluids (Sterofundin^®^ ISO) at a rate of 0.5 - 2.0 ml/kgBW/h under careful observation of the fluid balance.

### 2.3 Surgical treatment and C5/CD14 inhibition therapy

Surgical treatment and C5/CD14 inhibition therapy were applied after the 90-min shock phase. For the DCO group, fracture fixation of both femur fractures was performed using external fixators (Radiolucent Fixator, Orthofix US LLC, Texas, TX, USA), while the animals of the ETC and ETC + C5/CD14 inhibition group received intramedullary nailing. A T2^TM^ arthrodesis nailing system (Stryker^®^, Duisburg, Germany) was used. Considering the relatively short length of the porcine femora, this system was found to be well-suitable for this model. Reaming was performed before intramedullary nailing. The ETC + C5/CD14 inhibition group additionally received C5- and CD14 inhibiting medication. RA101295 (2-kDa peptide, UCB Pharma, Brussels, Belgium) was applied as an inhibitor of C5 cleavage. Furthermore, the recombinant anti-porcine CD14 antibody rMil2 (clone MIL2) was applied. This antibody was synthesized by the Mollnes’ group as reported elsewhere (24) and then produced on a larger scale, following GMP standards, by ExcellGene SA (Monthey, Switzerland).

The combined C5/CD14 inhibitory therapy was given intravenously as described in (20). To set up the working concentrations of the interventional drugs and an appropriate regimen of administration, the first animal in the ETC + CD5/CD14 group was given a C5 inhibition bolus of 3 mg/kgBW at 30 min after polytrauma induction, followed by a continuous infusion of 0.55 mg/kgBW/h for 64 h, and a CD14 inhibitor bolus of 5 mg/kgBW at 30 min after the injuries. Based on the obtained results, the administration of the drugs in the ETC + C5/CD14 inhibition group was adjusted as follows: C5 inhibitor bolus of 5 mg/kgBW at 30 min after the injuries, followed by a continuous infusion of 1.1 mg/kgBW/h for 72 h, and CD14 inhibitor boluses of 5 mg/kgBW at 30 min, 12 h, and 30 h after the injuries, and 2.5 mg/kgBW at 60 h after the injury. No treatment efficacy variability was observed between the first and other n = 3 animals in this treatment group. Therefore, the four animals were combined as part of the ETC + C5/CD14 inhibition group during data treatment and analysis.

In work performed previously by the Mollnes’ group, the dose of the CD14 inhibitory therapy was titrated to reach saturation in a pig model of E. coli sepsis (25) and has been used as a standard dose in later studies. For the C5 inhibitor, the tentative dose chosen was based on 3 pilot pigs observed for 8 h (unpublished data). The pharmacodynamics of the C5 therapy in pigs observed for 72 h were performed in this study (20) allowing for accurate dosage adjustment.

### 2.4 Animal model: vital parameters monitoring and confirmation of lung injury

Vital signs were recorded for the duration of the experiment that have been reported elsewhere (20, 26). Relevant to the study presented here, base excess in all polytrauma groups were on average median values 4 mmol/l, whereas the sham group showed levels of 8 mmol/l. Upon 24 h of intensive care, levels normalized to the sham levels. Similarly, lactate levels, averaging 1.8 mmol/l in the polytrauma groups decreased to sham levels of 0.4 mmol/l upon 24 h of intensive care (26). The cardiovascular monitoring showed an elevated heart rate in the polytrauma groups, that decreased after 24 h to median values of 67 BPM, still notably higher than the 41 BMP recorded in the sham animals (20). However, the MAP average of median values of 61 mmHg for the polytrauma groups approximated the 60 mmHg recorded for the sham animals at 24 h after the beginning of the experiment (20). Regarding tissue damage, the histological evaluation of the lung specimens confirmed the presence of injury in the ETC group that appeared diminished by the C5/CD14 inhibition. Of note, the wet/dry ratio of the lung in the C5/CD14 inhibition group was not significantly different from that of the sham animals (unpublished data). Saturation levels remained constant at over 97% from 1.5 h on until the end of the experiment.

### 2.5 Sample collection and tissue disruption

Lung samples were collected directly after sacrifice, snap-frozen in liquid nitrogen, and stored at -80°C. A Qiagen Tissue Lyser LT (Qiagen, Venlo, The Netherlands) was used for 5 min at 50 oscillations to disrupt the samples in Trizol Reagent (Thermo Fisher Scientific, Waltham, MA, USA). The homogenate was stored at -80°C for later use.

### 2.6 RNA isolation and cDNA synthesis

Chloroform-phenol RNA isolation was performed using GlycoBlue as a co-precipitant (Thermo Fisher Scientific). RNA quantity and purity was determined using spectrophotometry (Biodrop µLite+, Biochrom, Holliston, MA, USA) for which cut-off values of ≥ 1.7 and ≥ 1.8 were applied for the A260/A230 and A260/A280 ratios, respectively. If necessary, an RNA purification was performed with the Monarch^®^ RNA Cleanup Kit (New England Biolabs, Ipswich, MA, USA). Per animal, 40 ng of template RNA were transcribed to cDNA using the miRCURY LNA RT kit (Qiagen) according to the manufacturer’s instructions.

### 2.7 MiRNA qPCR array

Customized miRCURY miRNA qPCR arrays (Qiagen) were used to determine pulmonary miRNA expression profiles. In total, 91 miRNAs associated with pulmonary function, fibrosis, inflammation, and the post-traumatic immune response were evaluated. A list of the miRNA included in the customized panel, their sequence, and accession number can be found in **Table 1**. For qPCR array analysis, cDNA samples of each experimental group were pooled using equal amounts of cDNA from each sample. For accurate and reliable gene expression normalization, the geNorm normalization algorithm was used which calculates a normalization factor based on a variety of miRNAs.

**Table 1:** A list of the miRNA included in the customized panel, their sequence, and accession number.

miRNA	Target sequence	Qiagen Global Gene ID	Used in Validation Assay
hsa-let-7d-5p	AGAGGUAGUAGGUUGCAUAGUU	YP00204124	No
ssc-miR-1	UGGAAUGUAAAGAAGUAUGUA	YP02103615	No
hsa-miR-101-3p	UACAGUACUGUGAUAACUGAA	YP00204786	No
hsa-miR-107	AGCAGCAUUGUACAGGGCUAUCA	YP00204468	No
cfa-miR-10a	UACCCUGUAGAUCCGAAUUUGU	YP02113807	No
xtr-miR-10b	UACCCUGUAGAACCGAAUUUGU	YP02104576	No
**ssc-miR-122-5p**	**UGGAGUGUGACAAUGGUGUUUGU**	**YP02101912**	**Yes**
hsa-miR-125b-5p	UCCCUGAGACCCUAACUUGUGA	YP00205713	No
hsa-miR-126-3p	UCGUACCGUGAGUAAUAAUGCG	YP00204227	No
**hsa-miR-129-5p**	**CUUUUUGCGGUCUGGGCUUGC**	**YP00204534**	**Yes**
hsa-miR-132-3p	UAACAGUCUACAGCCAUGGUCG	YP00206035	No
mml-miR-133a	UUGGUCCCCUUCAACCAGCUG	YP02119318	No
sha-miR-141	UAACACUGUCUGGUAAAGAUG	YP02104712	No
gga-miR-142-3p	UGUAGUGUUUCCUACUUUAUGG	YP02114275	No
hsa-miR-143-3p	UGAGAUGAAGCACUGUAGCUC	YP00205992	No
xtr-miR-145	GUCCAGUUUUCCCAGGAAUCCCUU	YP00205962	No
hsa-miR-146a-5p	UGAGAACUGAAUUCCAUGGGUU	YP00204688	No
ssc-miR-146b	UGAGAACUGAAUUCCAUAGGC	YP02106323	No
hsa-miR-148a-3p	UCAGUGCACUACAGAACUUUGU	YP00205867	No
hsa-miR-150-5p	UCUCCCAACCCUUGUACCAGUG	YP00204660	No
ssc-miR-155-5p	UUAAUGCUAAUUGUGAUAGGGG	YP02103475	No
hsa-miR-15b-5p	UAGCAGCACAUCAUGGUUUACA	YP00204243	No
hsa-miR-16-5p	UAGCAGCACGUAAAUAUUGGCG	YP00205702	No
hsa-miR-17-5p	CAAAGUGCUUACAGUGCAGGUAG	YP02119304	No
ssc-miR-18a	UAAGGUGCAUCUAGUGCAGAUA	YP02119027	No
**hsa-miR-192-5p**	**CUGACCUAUGAAUUGACAGCC**	**YP00204099**	**Yes**
**ssc-miR-194a-5p**	**UGUAACAGCAACUCCAUGUGG**	**YP02113287**	**Yes**
hsa-miR-195-5p	UAGCAGCACAGAAAUAUUGGC	YP00205869	No
hsa-miR-196a-5p	UAGGUAGUUUCAUGUUGUUGGG	YP00204386	No
hsa-miR-199a-5p	CCCAGUGUUCAGACUACCUGUUC	YP00204494	No
ssc-miR-199b-5p	CCCAGUGUUUAGACUAUCUGUU	YP02106279	No
hsa-miR-19a-3p	UGUGCAAAUCUAUGCAAAACUGA	YP00205862	No
hsa-miR-19b-3p	UGUGCAAAUCCAUGCAAAACUGA	YP00204450	No
ssc-miR-155-3p	UCCUACAUGUUAGCAUUAACA	YP02108624	No
rno-miR-200b-3p	UAAUACUGCCUGGUAAUGAUGAC	YP00205111	No
ssc-miR-146a-3p	CCUGUGAAGUUUAGUUCUUCAG	YP02117141	No
hsa-miR-204-5p	UUCCCUUUGUCAUCCUAUGCCU	YP00206072	No
hsa-miR-208b-3p	AUAAGACGAACAAAAGGUUUGU	YP00204636	No
ssc-miR-20a-5p	UAAAGUGCUUAUAGUGCAGGUA	YP02102800	No
mmu-miR-31-5p	AGGCAAGAUGCUGGCAUAGCUG	YP00205159	No
hsa-miR-215-5p	AUGACCUAUGAAUUGACAGAC	YP00204598	No
hsa-miR-21-5p	UAGCUUAUCAGACUGAUGUUGA	YP00204230	No
ssc-miR-216	UAAUCUCAGCUGGCAACUGUGAG	YP02113182	No
cfa-miR-217	UACUGCAUCAGGAACUGAUUGGAU	YP02119707	No
rno-miR-223-3p	UGUCAGUUUGUCAAAUACCCC	YP00205120	No
hsa-miR-23a-3p	AUCACAUUGCCAGGGAUUUCC	YP00204772	No
hsa-miR-24-3p	UGGCUCAGUUCAGCAGGAACAG	YP00204260	No
hsa-miR-26a-5p	UUCAAGUAAUCCAGGAUAGGCU	YP00206023	No
bta-miR-26b	UUCAAGUAAUUCAGGAUAGGUU	YP00205953	No
hsa-miR-27a-3p	UUCACAGUGGCUAAGUUCCGC	YP00206038	No
hsa-miR-27b-3p	UUCACAGUGGCUAAGUUCUGC	YP00205915	No
ssc-miR-29a-3p	CUAGCACCAUCUGAAAUCGGUUA	YP02101718	No
hsa-miR-29b-3p	UAGCACCAUUUGAAAUCAGUGUU	YP00204679	No
hsa-miR-29c-3p	UAGCACCAUUUGAAAUCGGUUA	YP00204729	No
ssc-miR-301	CAGUCCAAUAGUAUUGUCAAAGC	YP02115322	No
hsa-miR-30a-5p	UGUAAACAUCCUCGACUGGAAG	YP00205695	No
hsa-miR-214-5p	UGCCUGUCUACACUUGCUGUGC	YP00204575	No
hsa-miR-28-3p	CACUAGAUUGUGAGCUCCUGGA	YP00204119	No
mmu-miR-324-5p	CGCAUCCCCUAGGGCAUUGGUGU	YP02119698	No
cfa-miR-325	CCUAGUAGGUGUUCAGUAAGUGU	YP02105608	No
mml-miR-32-5p	UAUUGCACAUUACUAAGUUGC	YP02119706	No
hsa-miR-328-3p	CUGGCCCUCUCUGCCCUUCCGU	YP00204364	No
ssc-miR-335	UCAAGAGCAAUAACGAAAAAUG	YP02112093	No
hsa-miR-338-3p	UCCAGCAUCAGUGAUUUUGUUG	YP00204719	No
hsa-miR-34a-5p	UGGCAGUGUCUUAGCUGGUUGU	YP00204486	No
hsa-miR-331-3p	GCCCCUGGGCCUAUCCUAGAA	YP00206046	No
hsa-miR-375-3p	UUUGUUCGUUCGGCUCGCGUGA	YP00204362	No
hsa-miR-374a-5p	UUAUAAUACAACCUGAUAAGUG	YP00204758	No
hsa-miR-378a-3p	ACUGGACUUGGAGUCAGAAGGC	YP00205946	No
**ssc-miR-382**	**AAGUUGUUCGUGGUGGAUUCG**	**YP02103633**	**Yes**
hsa-miR-423-3p	AGCUCGGUCUGAGGCCCCUCAGU	YP00204488	No
hsa-miR-423-5p	UGAGGGGCAGAGAGCGAGACUUU	YP00205624	No
hsa-miR-451a	AAACCGUUACCAUUACUGAGUU	YP02119305	No
hsa-miR-491-5p	AGUGGGGAACCCUUCCAUGAGG	YP00204695	No
dre-miR-140-3p	UACCACAGGGUAGAACCACGGAC	YP02111968	No
**mmu-miR-503-5p**	**UAGCAGCGGGAACAGUACUGCAG**	**YP00205094**	**Yes**
hsa-miR-425-3p	AUCGGGAAUGUCGUGUCCGCCC	YP00204038	No
hsa-miR-425-5p	AAUGACACGAUCACUCCCGUUGA	YP00204337	No
hsa-miR-455-5p	UAUGUGCCUUUGGACUACAUCG	YP00204363	No
hsa-miR-151a-5p	UCGAGGAGCUCACAGUCUAGU	YP00204007	No
hsa-miR-744-5p	UGCGGGGCUAGGGCUAACAGCA	YP00204663	No
hsa-miR-7-5p	UGGAAGACUAGUGAUUUUGUUGUU	YP02119317	No
ssc-miR-874	CUGCCCUGGCCCGAGGGACCGAC	YP02103300	No
hsa-miR-92a-3p	UAUUGCACUUGUCCCGGCCUGU	YP00204258	No
cel-miR-39-3p	UCACCGGGUGUAAAUCAGCUUG	YP00203952	No
cel-miR-39-3p	UCACCGGGUGUAAAUCAGCUUG	YP00203952	No
hsa-miR-99a-5p	AACCCGUAGAUCCGAUCUUGUG	YP00204521	No
ssc-miR-151-3p	CUAGACUGAAGCUCCUUGAGGA	YP02108125	No
mmu-miR-202-5p	UUCCUAUGCAUAUACUUCUUU	YP00205654	No
hsa-miR-708-5p	AAGGAGCUUACAAUCUAGCUGGG	YP00204490	No
hsa-miR-22-3p	AAGCUGCCAGUUGAAGAACUGU	YP00204606	No
hsa-miR-22-5p	AGUUCUUCAGUGGCAAGCUUUA	YP00204255	No
hsa-miR-542-3p	UGUGACAGAUUGAUAACUGAAA	YP00205444	No
**UniSp6**	**CUAGUCCGAUCUAAGUCUUCGA**	**YP00203954**	**Yes**
UniSP3	CATCGATTGTACTAGGCTACGTTTTTTTTT	YP02119288	No
UniSP3	CATCGATTGTACTAGGCTACGTTTTTTTTT	YP02119288	No

The validated miRNAs are indicated by using bold text in a grey highlighted row.

The miRNA expression levels of the arrays were assessed by the cycle threshold (Ct) value using the CFX96 Real-Time PCR system (Bio-Rad, Hercules, CA, USA). Fold regulations were calculated using the 2^−ΔΔCt^ method; a downregulation was represented as the negative inverse of the acquired 2^−ΔΔCt^ value. A fold regulation of ≥ 2 or ≤ -2 was considered significantly deregulated. Melting curve analysis was conducted along with a specified cut-off Ct value of 35, beyond which miRNAs were not taken into consideration for further analysis.

### 2.8 MiRNA validations

MiRNAs that resulted significantly deregulated from the array analyses were validated in each individual sample using miRCURY qPCR primer assays. Expression levels were assessed by the cycle threshold (Ct) value using the CFX96 Real-Time PCR system (Bio-Rad). U6 snRNA was chosen as a housekeeping gene. An overview of the validated miRNAs along with details on sequence and accession number can be found, highlighted in grey, in **Table 1**. Fold changes were calculated using the 2^−ΔΔCt^ method. Melting curve analysis was conducted along with a specified cut-off Ct value of 35, beyond which miRNAs were not taken into consideration for further analysis.

### 2.9 *In silico* mRNA target prediction

Genes of interest were screened through an extensive literature review, focusing on the (post-traumatic) inflammatory response, alveolar-capillary barrier disruption, fibrosis, immune cell infiltration, and pathways involved in cellular survival, communication and proliferation, and tissue remodeling. The target prediction analyses were performed with miRanda target scanner version 3.3a (http://www.microrna.org), which bases its target prediction on sequence complementarity based on position-weighted local alignment, free energy of the duplex structure, and evolutionary conservation of a target site (27). MiRNA-mRNA interactions were depicted using Cytoscape 3.10.0 (http://www.cytoscape.org/).

### 2.10 Bioinformatics analyses

R software (Version 4.3.0; packages “clusterProfiler” and “pathview”) was used to conduct Gene Ontology (GO) and Kyoto Encyclopedia of Genes and Genomes (KEGG) enrichment analyses based on the predicted target genes to further investigate the function of those genes (28, 29). A hypergeometric distribution test was used to determine statistical significance, and a *p* < 0.05 was considered statistically significant. A significantly enriched term in GO and KEGG analyses refers to a biological term or pathway of which a great number of the predicted mRNA targets are involved in, indicating its potential relevance to the biological context under investigation. The enrichment score was calculated as the negative logarithm of the *p*-value to represent the overrepresentation degree of a specific GO or KEGG term in the target gene set compared to the reference set.

### 2.11 Statistical analysis

Statistical analyses were performed with GraphPad Prism version 9.1.1 (GraphPad Software, San Diego, CA, USA). Data are presented as mean or median, accompanied by the standard deviation or quartiles as applicable. Normality testing was performed using the Shapiro-Wilk test, after which one-way ANOVA or Kruskal-Wallis tests were applied as appropriate. For this study, a *p* < 0.05 was considered statistically significant.

## 3 Results

### 3.1 MiRNA expression profile

**Figure 1:**
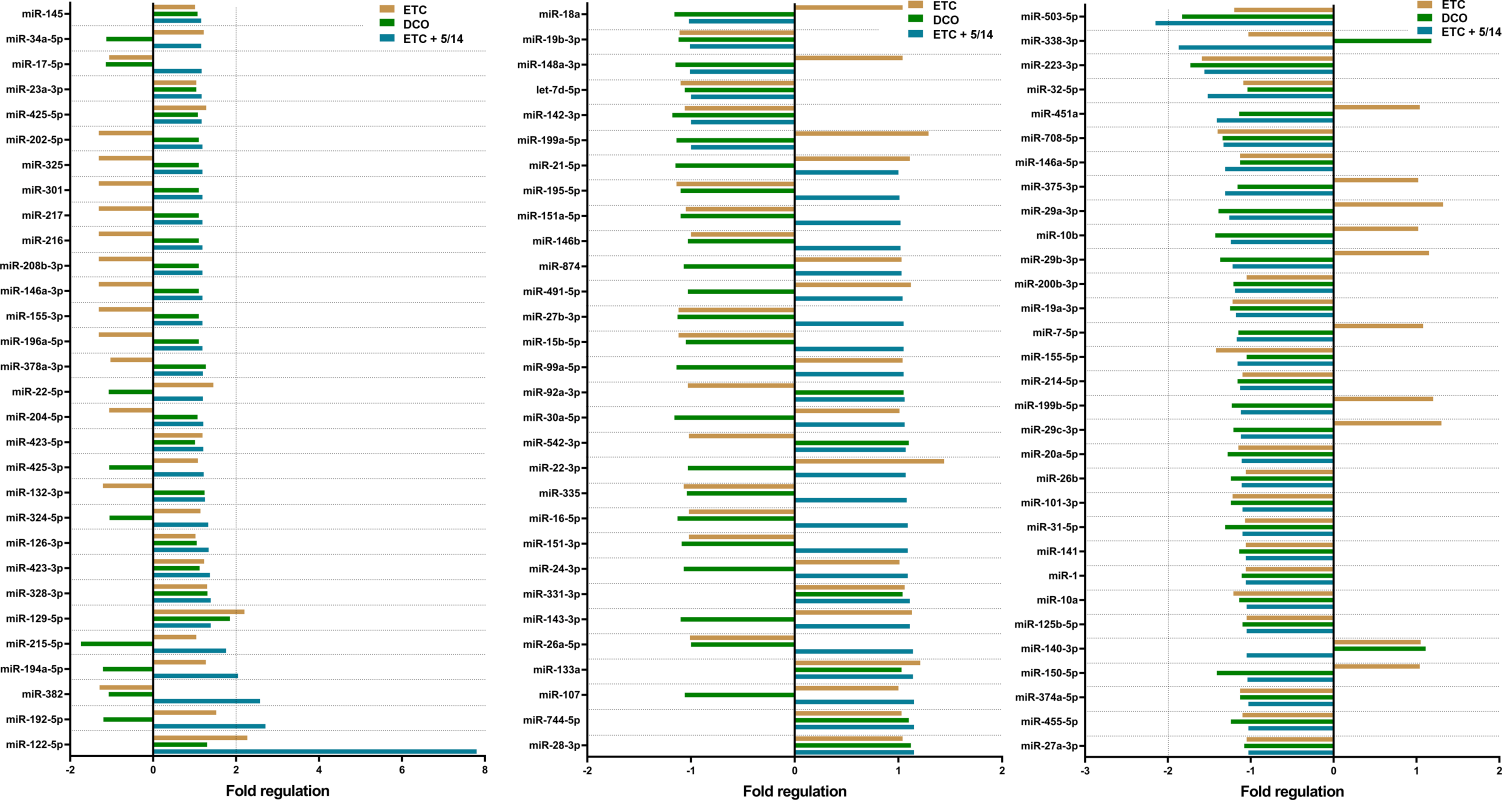
Pulmonary microRNA expression signature of pooled samples of the Early Total Care (ETC), Damage Control Orthopedics (DCO), and ETC + C5/CD14 inhibition (5/14) groups as detected by the custom made, Qiagen qPCR array. Results were normalized using Qiagen’s geNorm algorithm and depicted as fold regulation. Vertical dotted lines display the threshold of an absolute difference of 2-fold regulations, above or below which microRNAs were considered to be significantly deregulated.

All 91 miRNAs of interest were detected in the evaluated samples. Among them, six miRNAs showed a significant deregulation (**Figure 1**). The array data indicates that miR-122-5p and miR-129-5p were upregulated in the ETC group compared to the sham group. No significant deregulation was observed in the DCO group for any of the screened miRNAs. In the ETC + C5/CD14 inhibition group, miR-122-5p, miR-192-5p, miR-382, and miR-194-5p were upregulated, while miR-503-5p was downregulated in comparison to the sham group (**Figure 1**). These six miRNAs were selected for further individual validation based on their observed deregulations, as well as their predicted implications in post-traumatic pulmonary pathophysiology.

### 3.2 MiRNA validations

**Figure 2:**
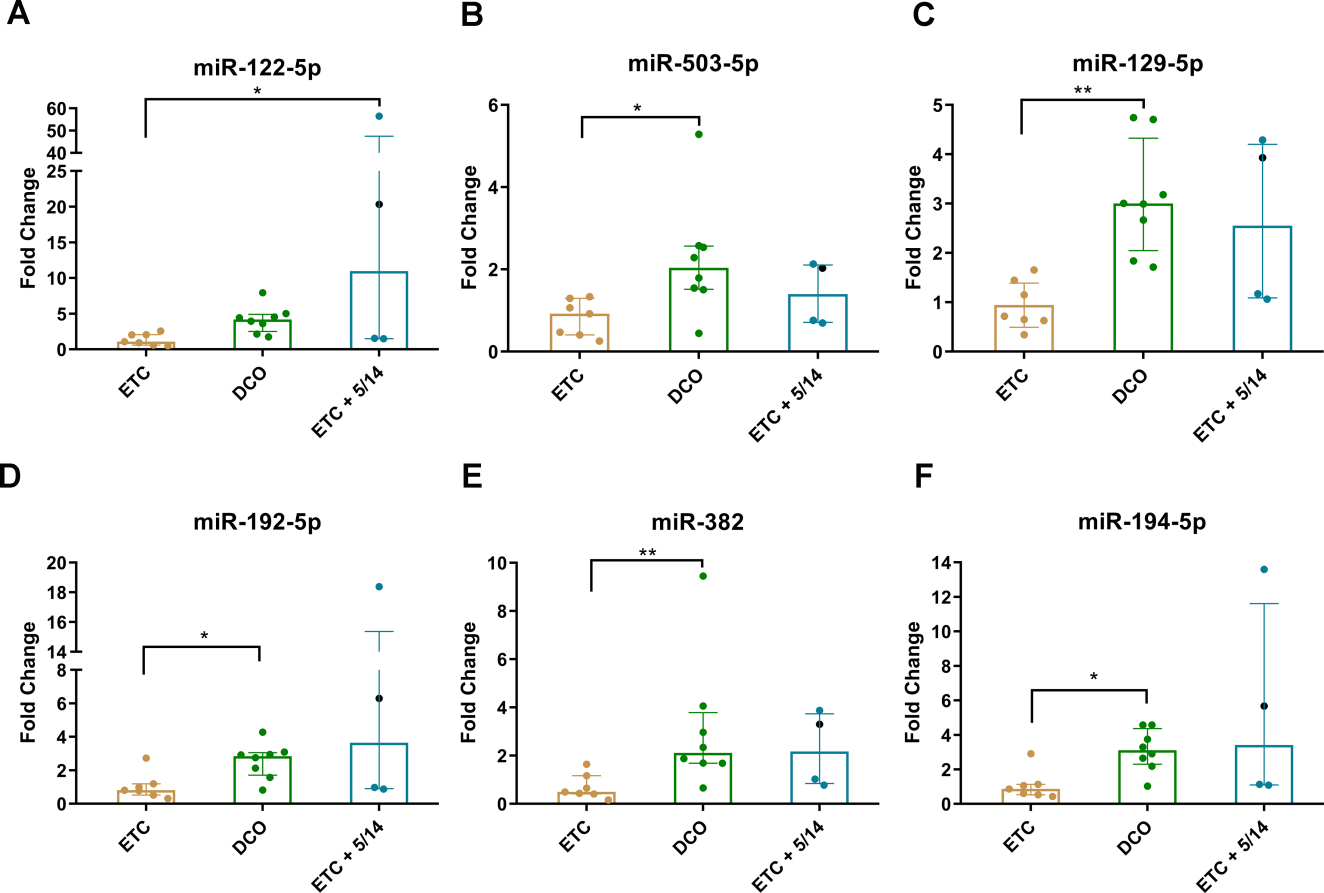
Validation of the six significantly deregulated miRNAs which were identified in the pooled miRNA qPCR array analyses for each treatment group: **(A)** miR-122-5p; **(B)** miR-503-5p; **(C)** miR-129-5p; **(D)** miR-192-5p; **(E)** miR-382; **(F)** miR-194-5p. Columns show median (horizontal bars), the 25th and 75th percentiles (error bars). In addition, individual values corresponding to each animal have been added as colored dots * *p* < 0.05, ** *p* < 0.01. Of note, the first animal in the ETC + C5/CD14 inhibition (5/14) group treated following a slightly different C5/CD14 therapy routine has been specifically marked with a black dot.

The expression levels for the six validated miRNAs were lowest in the ETC group as compared to the other treatment groups. In the DCO group, five of the six miRNA expression levels resulted in significant upregulation compared to the ETC group. These were miR-503-5p (*p* < 0.05), miR-129-5p (*p* < 0.01), miR-192-5p (*p* < 0.05), miR-382 (*p* < 0.01), and miR-194-5p (*p* < 0.05) (**Figures 2B–F**). Interestingly, the expression levels of miR-503-5p, miR-129-5p, and miR-382 in the ETC + C5/CD14 inhibition group showed no statistically significant differences from those obtained for the DCO group (*p* > 0.05), with only a relatively small variance appreciated. The expression of the other three miRNAs, miR-122-5p, miR-192-5p, and miR-194-5p, showed higher median values compared to the DCO group, but these were associated with increased variances. Lastly, miR-122-5p was significantly upregulated in the ETC + C5/CD14 inhibition group as compared to the ETC group (*p* < 0.05) (**Figure 2A**).

### 3.3 *In silico* target prediction

**Figure 3:**
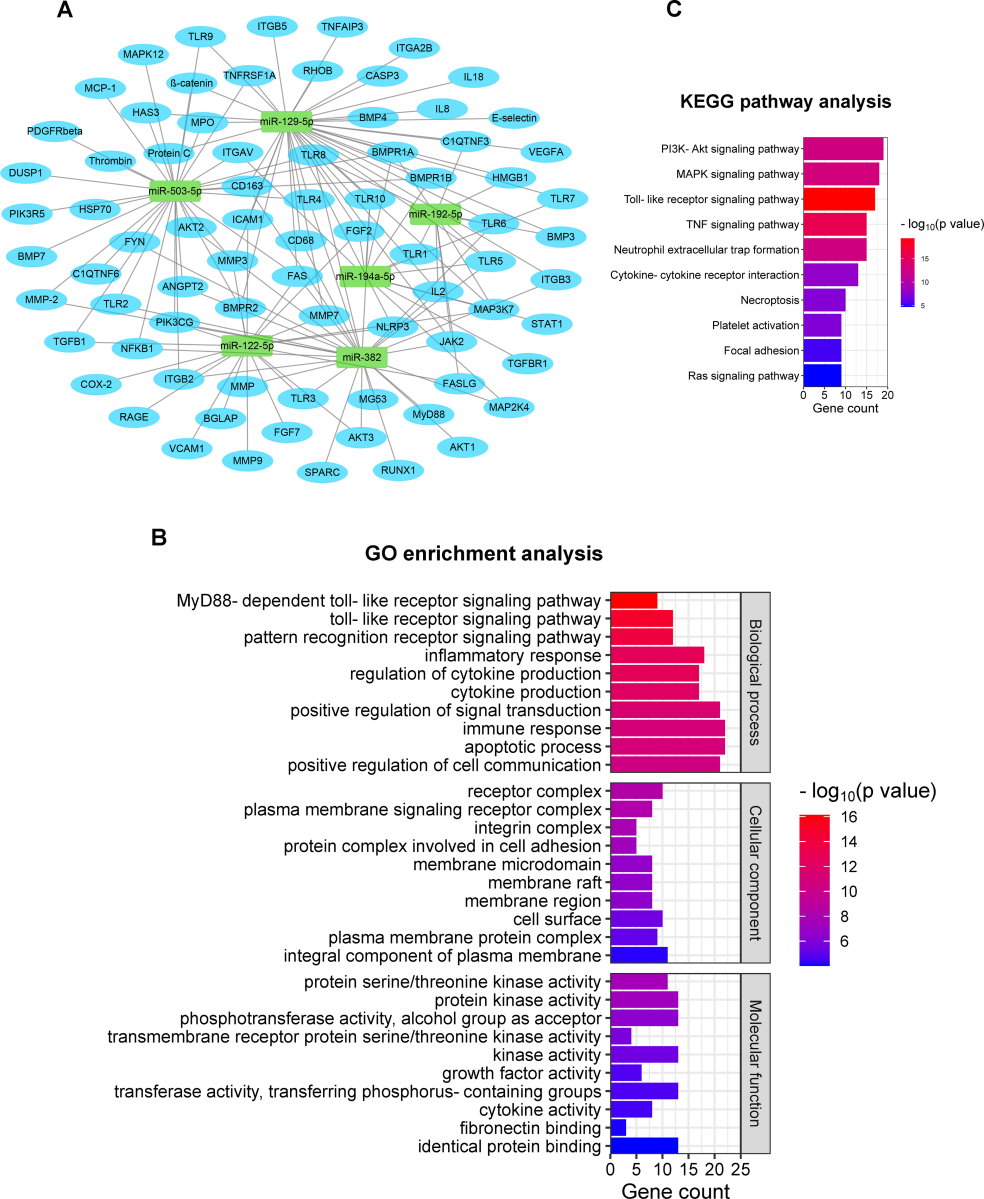
*In silico* prediction of mRNA targets of the validated miRNAs, including GO & KEGG enrichment analyses. **(A)** Target gene prediction of the six validated miRNAs; **(B)** 10 most enriched GO terms are listed in three categories based on biological process, cellular component, and molecular function [ranking by -log_10_(*p*-value)]; **(C)** KEGG pathway terms related to respiratory failure after polytrauma (ranking by number of enriched genes). GO, Gene Ontology; KEGG, Kyoto Encyclopedia of Genes and Genomes.

The mRNA target prediction with the miRanda algorithm revealed a total of 78 mRNA targets for the six validated miRNAs. Targets were involved in several biomolecular pathways relevant to this polytrauma scenario. These were, among others, inflammation, cellular adhesion, fibrosis, and immune cell activation and functioning. Notably, a large number of predicted targets showed implications in the post-traumatic immune response, such as IL-1β, IL-2, IL-8, and IL-18, as well as TNF-α, and immune cell markers like CD68 and CD163. An overview of the results from the *in silico* target prediction analysis is shown in **Figure 3A**.

### 3.4 GO and KEGG enrichment analyses

Comprehensive functional enrichment analyses, including GO and KEGG analyses, were performed to further investigate the biological implications of the predicted mRNA targets in relation to this study.

The GO enrichment analysis revealed a wide range of enriched biological processes, cellular components, and biomolecular functions associated with the miRNA target genes. The 10 most significant GO terms from each category were selected by ranking the Enrichment Score [-log_10_(*p*-value)] for display (**Figure 3B**). In terms of biological processes, we identified significantly enriched terms related to, among others, MyD88-dependent toll-like receptor, toll-like receptors, and pattern recognition receptors, highlighting the involvement of miRNAs in DAMP and pathogen-associated molecular pattern (PAMP) recognition and signaling. Additionally, an enrichment in GO terms associated with the inflammatory response, cytokine production, signal transduction, immune response, and apoptosis, was observed, indicating the involvement of miRNAs in respiratory failure. Various of the miRNA target genes were found to be implicated with several cellular components, such as receptor complex, plasma membrane, membrane raft, and cell surface, implicating a regulatory role of miRNAs in intracellular signaling. Regarding biomolecular functions, the mRNA targets demonstrated significant enrichments in GO terms associated with the activity of several kinases, growth factors, and cytokines. These findings further endorse the involvement of miRNAs in the modulation of various signaling pathways and cytokine cascades important in the post-traumatic pulmonary response.

Furthermore, KEGG analyses provided valuable insights into the potential biomolecular pathways influenced by the miRNA targets. According to the gene ranking based on statistical significance and the possible correlation with polytrauma pulmonary complications, 10 KEGG pathway terms were selected (**Figure 3C**). The enrichment pathways were observed in PI3K-Akt signaling pathway, MAPK signaling pathway, toll-like receptor signaling pathway, TNF signaling pathway, and Ras signaling pathway. These results show the involvement of miRNAs in critical cellular signaling cascades after trauma. Moreover, the enrichment terms of neutrophil extracellular trap formation, cytokine-cytokine receptor interaction, necroptosis, platelet activation, and focal adhesion revealed the crucial role of miRNAs in pathophysiological immunological processes after trauma, including neutrophil recruitment, cytokine activation, cell necrosis, and coagulopathy.

## 4 Discussion

This study profiled post-traumatic pulmonary miRNA expression patterns in a translational polytrauma animal model. In this model, two surgical treatment strategies (i.e., ETC and DCO) and a novel C5/CD14 inhibition therapy were applied to determine their effects on common pulmonary complications after polytrauma.

### 4.1 Pulmonary miRNA expression patterns: comparing ETC and DCO

It is known that intramedullary reaming can contribute to pulmonary complications in severely polytraumatized patients, such as acute lung injuries or an aggravation of lung contusion (30). In this study, surgical invasiveness had a significant impact on the post-traumatic pulmonary miRNA expression signature, demonstrating a systemic effect of a locally applied fracture fixation strategy. This was supported by the differential upregulation of miR-503, miR-129, miR-192, miR-382, and miR-194 in the DCO group.

During intramedullary reaming in ETC, substances such as bone marrow intravasate due to increased intramedullary pressure, causing pulmonary fat embolisms (31). These pulmonary fat embolisms can cause ischemic necrosis and edema of lung tissue, and also promote the expression of pro-fibrotic agents, such as collagen and smooth muscle actin (32, 33). MiR-503 reduces the migration and invasion of fibroblasts in the pulmonary interstitium and decreases the expression of fibrotic factors by suppressing FGFR1 and VEGFA (34). It also limits alveolar epithelial-mesenchymal transition by targeting PI3K p85, leading to a delayed progression into the fibrotic phase of ARDS (35). Furthermore, miR-503 was predicted to target several matrix metalloproteinases (MMPs), which are a family of endopeptidases that degrade and reorganize the extracellular matrix (ECM) (36). In the early exudative phase of ARDS, the ECM-mediated integrity of the alveolar epithelial-endothelial structure is disrupted due to the activation of these MMPs (37). The upregulation of miR-503 in the DCO group may, thus, contribute to a preservation of epithelial-endothelial functioning. Furthermore, its upregulation can contribute to the maintenance of the alveolar-capillary barrier, thereby decreasing alveolar edema formation in ARDS.

The other two miRNAs that were upregulated in the DCO group were miR-192 and miR-382. These two miRNAs exhibit anti-inflammatory effects by targeting, among others, IL-1β, IL-6, and TNF-α (38, 39). Furthermore, they inhibit the polarization of macrophages towards the pro-inflammatory M1 phenotype (40, 41). In turn, research has shown that M1 macrophages can cause alveolar edema upon stimulation by pro-inflammatory cytokines, such as after polytrauma (42). Looking at these miRNA expression patterns, reduced surgical invasiveness seems to promote an anti-inflammatory, lung-protective microenvironment. In terms of mRNA target prediction, miR-382 targeted angiopoietin 2, which plays a key role in disrupting the pulmonary endothelial barrier (43). Another target gene of miR-382 is the integrin subunit ITGB3, which under normal circumstances promotes the proliferation of pulmonary fibroblasts, thereby causing more pulmonary fibrosis (44). Hence, the upregulation of miR-382 in the DCO group could contribute to maintaining a functional pulmonary endothelial barrier and alleviate post-traumatic inflammation and fibrosis in the lung.

Another key factor that contributes to the development of ARDS is hemorrhage-induced apoptosis and necrosis of immune and alveolar epithelial cells (45). Interestingly, miR-129 and miR-194 elicit anti-apoptotic as well as anti-inflammatory effects *in vitro* (46, 47), and were both upregulated in the DCO group. Reduced perioperative blood loss in DCO may be an important factor that underlies the upregulation of these miRNAs. Apart from anti-apoptotic and anti-inflammatory effects, upregulations of both miR-129 and miR-194 enhanced alveolar epithelial cell viability in several LPS-induced acute lung injury models (48, 49). The expression patterns of these miRNAs therefore open new doors to biomolecular pathways that could contribute to decreased pulmonary complications after DCO. Cellular invasion in injured and/or inflamed sites is an important aspect of the post-traumatic immune response. IL-2, IL-8, and IL-18 were found among the predicted mRNA targets of miR-129. These interleukins are known to stimulate the recruitment and infiltration of neutrophils and promote pulmonary inflammation (50). Furthermore, ICAM-1 and E-selectin, both targeted by miR-129, are excessively expressed on pulmonary endothelial cell surfaces during inflammatory stimulation and mediate leucocyte adhesion and migration across the pulmonary endothelium (51, 52). Therefore, the increased expression of miR-129 in the DCO group may aid in reducing inflammatory cell infiltration in the lung parenchyma. In addition, miR-129 and miR-194 were predicted to target FGF2 and VEGFA, which can promote alveolar fibrosis through collagen synthesis of fibroblasts and myofibroblasts (53). VEGF can increase microvascular permeability when interacting with the VEGFR, which can lead to alveolar edema (54). Both, miR-129 and miR-194, were upregulated in the DCO group and should suppress FGF2 and VEGFA, subsequently reducing alveolar fibrosis and edema in ARDS.

Overall, the pulmonary miRNA signature of the DCO group was more anti-inflammatory and tissue regenerative in nature, as compared to that of the ETC group.

### 4.2 Combining ETC with C5/CD14 inhibition - effect on the pulmonary miRNA signature

Although ETC aims at reducing immobilization times for the patient and requires fewer follow-up surgeries, it is associated with complication risks in unstable polytrauma patients, among which pulmonary fat embolisms and respiratory failure are of relevance. Developing therapeutic tools that could mitigate the negative impacts of surgical invasiveness associated with ETC is therefore of great importance. This study examined the influence of adding a novel C5/CD14 inhibition therapy to the regular ETC treatment algorithm and investigated its influence on pulmonary miRNA expression patterns.

Relevantly, the distinctive upregulation of miR-192 and miR-194 in the ETC + C5/CD14 inhibition group may be responsible for a protective microenvironment by eliciting anti-inflammatory, anti-apoptotic, and anti-fibrotic effects. In the early pathogenesis of acute lung injury, alveolar epithelial cells, alveolar macrophages, and dendritic cells act as sensors to detect injury signals through Pattern Recognition Receptors (PRR). They initiate innate immune responses, such as the recruitment of leukocytes, secretion of proinflammatory cytokines and chemokines, and thereby lead to enhanced inflammation (55). MiR-192 and miR-194 suppress a wide range of proinflammatory mediators, inhibit M1 macrophage polarization, and reduce leukocyte recruitment in acute lung injury. The observed upregulation of these miRNAs as a result of combining ETC with C5/CD14 inhibition therapy contributes to a more balanced immune response after trauma and surgery.

MiR-122 demonstrated a significantly increased expression in the ETC + C5/CD14 inhibition group compared to the ETC group and, although not significant, was also upregulated compared to the DCO group. An overexpression of miR-122 has been associated with exacerbated oxidative stress and inflammation in an LPS-induced acute lung injury model (56, 57). However, miR-122 was also predicted to target several inflammatory mediators, such as COX2 and Fas ligand. Furthermore, the expression of COX2 and Fas ligand can be induced by complement factor C5 (58, 59). Additionally, both miR-122 and complement C5a were involved in PI3K/AKT signaling, which is key for apoptosis, oxidative stress, and neutrophil infiltration (60, 61). This suggests that the inhibition of these inflammatory mediators and signaling pathways by C5 inhibition may in part be achieved by increasing the expression of miR-122.

The mRNA target prediction analyses identified CD68 and CD163 as targets of miR-382. Interestingly these macrophage markers have been associated with inflammation and tissue damage (62, 63) as well as enhanced profibrotic factor expression (64). It might be interesting to investigate the combinatory effect of blocking CD14 along with the upregulation of miR-382. Among the predicted mRNA targets of miRNA-503, miRNA-129, miRNA-192, miRNA-382, and miRNA-194 were several TLRs and TLR-adaptor MyD88. The upregulation of these five miRNAs contributes to blocking LPS-induced PAMP and other TLR-related PRRs, thereby reducing inflammation. Stimulating this upregulation might be worth investigating in polytrauma scenarios.

Of note, a limitation of this study is that the sham group received mechanical ventilation, which does not mimic physiological conditions. This means that, although the observed differences in miRNA expression were treatment-specific, some miRNAs that may contribute to ventilator-induced lung injury are left unidentified due to the nature of this study. Moreover, the single injuries or the hemorrhagic shock could not be investigated alone as this would be logistically not feasible. Therefore, the exact contribution of the injuries or the hemorrhagic shock cannot be distinguished. However, we are interested in the effect of all components present during the polytrauma so that the miRNA expression results are important in this context. For the same reasons, the sole effect of the blood transfusion cannot be determined. Furthermore, the reduced sample size of the C5/CD14 inhibition group, given by the nature of this pilot study and drug supply limitations, is also considered a limitation of this study. To promote the bench-to-bedside transfer of this novel therapeutic agent, further research with a larger subject population over a longer period is required. Based on the encouraging results obtained in our pilot study, future research on the utility of this drug is guaranteed.

## 5 Conclusions

This study reports the first characterization of pulmonary miRNA expression after polytrauma in a translational animal model. In addition, different surgical and pharmacological treatment strategies were compared and reported here. Treatment-specific miRNA expression patterns were identified which depended on surgical invasiveness as well as the C5/CD14 inhibition therapy. MiRNA expression and bioinformatics analyses showed that the pulmonary miRNA fingerprint of the DCO group, as well as that of the ETC + C5/CD14 inhibition group, revealed distinct anti-inflammatory, more regenerative expression patterns, showing that reducing surgical invasiveness and immune modulation can be effective strategies to minimize complications in the context of severe polytrauma due to the second hit caused by surgical trauma.

This study provided important biomolecular insights into the underlying causes of post-traumatic pulmonary dysfunction and ARDS, and showed that surgical invasiveness and C5/CD14 inhibition therapy are important in steering tissue regenerative processes after polytrauma. In particular, it was shown that the combination of ETC and C5/CD14 inhibition therapy may enable the earlier application of definitive fracture fixation strategies in the acute post-traumatic phase by reducing the risk of associated pulmonary complications, such as ARDS. More research is needed to explore the role of miRNAs in injury-injury crosstalk after polytrauma, and in particular the role and involvements of miRNAs in common pulmonary complications after trauma.

## Data Availability

The datasets presented in this study can be found in online repositories. The names of the repository/repositories and accession number(s) can be found below: doi.org/10.34894/AHA6GU.
